# Optimization and evaluation of astragalus polysaccharide injectable thermoresponsive *in-situ* gels

**DOI:** 10.1371/journal.pone.0173949

**Published:** 2017-03-28

**Authors:** Zugong Yu, Fanxi Guo, Yangyang Guo, Zhenrui Zhang, Feng Wu, Xiaoqing Luo

**Affiliations:** Laboratory of Veterinary Pharmacology and Toxicology, College of Veterinary Medicine, Nanjing Agricultural University, Nanjing, Jiangsu Province, China; Universidade Estadual Paulista Julio de Mesquita Filho, BRAZIL

## Abstract

The objective of this study was to develop an injectable in situ forming gel system based on Poloxamer for sustained release of Astragalus polysaccharide (APS), thus achieved once or twice administration instead of frequent dosing during long-term treatment. The optimal formulation is 10 g APS, 18 g poloxamer 407, 2 g poloxamer 188, 0.15 g CMC-Na, 0.85 g sodium chloride in 100 ml gel in situ which had a preferable sol-gel transition temperature(*T* sol-gel) (34.1 ± 0.4°C), and good stability. In vitro release studies, all formulations containing polymer additives had prolonged release time and decreased initial burst to some extent. The optimal formulation containing 0.15% CMC-Na showed a best sustained release profile for about 132 h with the lowest initial burst in vitro about 16.30% in 12 h). In vivo, Male BALB/c mice (18–20 g) were administrated with APS in-situ gel just once, the values of immune organ indices, spleen lymphocyte proliferation, and serum IgM, IgG, IL-2 and IL-6 had significant increase, which was consistent with the mice given daily APS injections (7 times), while the above indices were increased more significantly in which administrated with APS in-situ gel twice. Based on these results, it can be concluded that the Poloxamer depot is a promising carrier for the sustained release of APS with an ideal release behavior.

## Introduction

Radix Astragali is one of the most important and popular medical herbs in traditional Chinese medicine [1]. Astragalus polysaccharides (APS), which is extracted from radix Astragali, is widely used in veterinary clinics against bacterial infections, viral infections, and other immune diseases [2–5].

Thus far, APS dosages have come to the veterinary clinical in the form of granules, powder, solution and fluid for injection, utilizing oral and injection delivery. However, delivery by injection has distinct advantages, because the polysaccharide in the oral doses can be destroyed by enzymes, resulting in low bioavailability[6, 7]. However, due to the short elimination half-life, it is necessary for APS injections to be frequently administrated to enhance immunity. For maximum immune-enhancing efficacy, the recommended use of APS injection in the Chinese Veterinary Pharmacopoeia was once a day for 7 consecutive days. Repeat injections not only consume human and material resources, but also result in stress to animals. Stress can retard positive outcomes, worsen illnesses and leave animals vulnerable to new diseases. Finding more effective delivery systems for APS, with significant sustained release effects and good compatibility with the drug itself, is a high-priority task.

Among the novel sustained-release preparations, thermosensitive in-situ forming gels may be one way to solve these problems. Thermosensitive in-situ forming gels use specific polymers which displays low viscosity at ambient temperature but a sharp viscosity increase following a certain temperature raise, producing a semi-solid gel at body temperature [8]. Thermosensitive in situ ge1 seem to be one of the most promising ones for the development of injectable drug delivery systems, which displays low viscosity at ambient temperature but a sharp viscosity increase following a certain temperature raise, producing a semi-solid gel at body temperature.The gel is a liquid at lower temperatures and only becomes a gel on above a certain temperature, known as a ‘sol-gel transition temperature’ [9]. One well-known range of polymers possessing thermoresponsive behavior is Poloxamers. Poloxamers are a triblock co-polymer poly(ethylene oxide)-b-poly(propylene oxide)-b-poly(ethylene oxide) (PEO-PPO-PEO) whose amphiphilic properties are due to hydrophilic ethylene oxide domains and hydrophobic propylene oxide domains [8]. They are widely used as a drug delivery system because of their physiological tolerability, low toxicity and good biocompatibility, ease of preparation, and good compatibility both with other drugs and pharmaceutical excipients [10].

The objectives of this study were to optimize the formulation of thermoresponsive APS in-situ gels, to evaluate their physicochemical properties, measure their in-vitro release rates, determine the effect of autoclaving on their physicochemical properties. In order to validate the sustained release effects in vivo, the immune-enhancing activities of APS in-situ forming gel were also measured in mice. The ultimate goal of this investigation was to achieve a prolonged duration of therapeutically effective drug concentration in the body, thus achieved once or twice administration instead of frequent dosing during long-term treatment.

## Materials and methods

### Materials and reagents

Radix Astragali was purchased from Inner Mongolia Qingfeng Pharmaceutical Co., Ltd. (Hohhot, China). A commercial formulated APS injection containing 20 mg APS per ml (with glucose meter) was obtained from Shandong Tianbang Biological Technology Co., Ltd. (Dezhou, China). Poloxamers (P407, P188) were purchased from BASF (Ludwigshafen, Germany). Carboxymethylcellulose sodium (CMC-Na) were purchased from Anhui Sunhere Pharmaceutical Excipients Co., LTD. (Anhui, China). RPMI 1640 was purchased from Gibco (Invitrogen Corporation, USA). 3-(4,5-Dimethylthiazol-2-yl)-2,5-diphenyltetrazoliumbromide (MTT), Dimethyl sulfoxide (DMSO) and lipopolysaccharide (LPS) were purchased from Sigma Co., USA. Cytoxan (CTX) was purchased from Jiangsu Hengrui Medicine Co., Ltd. (Lianyungang, China). Assay kits for interleukin-2 (IL-2), IL-6, immunoglobulins G (IgG) and IgM were all obtained from Nanjing Jiancheng Bioengineering Institute (Nanjing, China). All other chemicals used were of analytical reagent grade and obtained from Sinopharm Chemical Reagent Co., Ltd. (Shanghai, China).

### Preparation of APS solution

Radix Astragali (1000 g) was soaked in 8000 ml water for 4 h, decocted with the same volume of water three times for 60, 30 and 30 min in turn. The physical liquor was filtered through two-layered gauze, concentrated to 500 ml, cooled to ambient temperature, centrifuged for 20 min at 2500 rpm, and adjusted to a 60% ethanol concentration (v/v). After standing for 48 h, the physical liquor was filtered once more through two-layered gauze, put through a vacuum-rotary evaporation procedure to remove the ethanol, and concentrated into 125ml. APS were purified firstly through eliminating protein by trichloroacetic acid method, and then the APS was dissolved into 50 mg/ml with distilled water, added into a chromatographic column of DEAE-Cellulose-52 (2.5 cm × 30 cm) and eluted with distilled water [11]. The flow rate was maintained at 1 ml/min. The eluent was collected by an automatic fraction collector, 5 ml per tube. The carbohydrate concentration (%) of APS[calculated based on glucose (C_6_H_12_O_4_)] was determined by the phenol-sulfuric acid method, and the glucose read in 490 nm [12]. The protein content was determined by the Bradford method, and BSA was used as the standard material [11].

### Preparation of APS in-situ gels

The formulations of APS in-situ gel, containing various gelling agents, are shown in Table 1 The optimal dosage of each ingredient was determined by characterization of the APS in-situ gel. They were prepared by the cold method described by Wei et al [13]. Briefly, P407, P188 (in the case of formulations containing P188) and polymer additives were completely dissolved in cold saline. Then concentrated APS solution was added to the solutions of gelling agents and stirred continuously until homogeneous solutions were obtained. All samples were analyzed spectrophotometrically in triplicate to ensure content uniformity at a wavelength of 490 nm (UV-Visible Spectrophotometer, Hitachi U-2000, Japan). An APS content [calculated based on glucose (C_6_H_12_O_4_)] of 100 ± 10% of the labeled dosage was deemed acceptable.

**Table 1:** Formulation compositions in thermoresponsive APS in-situ gels.

Content of ingredients in each formulation(%, w/v)	Ingredients					
	APS	P407	P188	CMC-Na	NaCl	Water for injection ad
P407-16	10	16	0	0	0.85	100
P407-18	10	18	0	0	0.85	100
P407-20	10	20	0	0	0.85	100
P407-18+P188-1	10	18	1	0	0.85	100
P407-18+P188-1.5	10	18	1.5	0	0.85	100
P407-18+P188-2	10	18	2	0	0.85	100
P407-18+P188-2.5	10	18	2.5	0	0.85	100
P407-18+P188-2+C-0.1	10	18	2	0.1	0.85	100
P407-18+P188-2+C-0.15	10	18	2	0.15	0.85	100
P407-18+P188-2+C-0.2	10	18	2	0.2	0.85	100

### In vitro characterization

#### Gelation temperature and time determination

Gelation temperatures of the gels were measured using the test tube inverting method described in former studies [14]. 2 ml of the gel was transferred to a test tube, sealed with Parafilm, and immersed in a water bath at 4°C. The temperature of the bath was increased in increments of 1°C and left to equilibrate for 15 min at each new setting. The samples were examined for gelation, which is considered to have occurred when the meniscus no longer moves when tilted more than 90°. All measurements were performed in triplicate. The samples that could flow freely at 25 ± 1°C but not at 34 ± 1°C, and which had short sol-gel transition times, were accepted as thermoresponsive in-situ gels for this study.

#### Viscosity determination and pH determination

Viscosity of the samples was determined using a rotary viscosimeter and measured at a temperature of 25°C. The pH range of in-situ gel formulations should be between 6.0 and 7.5 to avoid irritation to the animal upon administration of the formulation. The pH value was determined using a pH meter (Hanna instruments 8417, USA). The formulation was maintained at 25°C and all measurements were performed in triplicate [15].

#### Autoclaving sterilization

Three batches of APS in-situ gels(Lot numbers: 01, 02, 03) were treated under the autoclaving sterilization conditions recommended by the Chinese Veterinary Pharmacopoeia. Briefly, vials containing 10 g of APS in-situ gel were placed in an autoclave (Hirayama, Japan) and exposed to steam at 121°C, at a pressure of about 15 psi, for 20 min. We then evaluated their viscosity, % LA labeled amount of APS, calculated based on glucose (C_6_H_12_O_4_)], sol-gel transition temperature(*T* sol-gel), and transition time. The data were to be compared to those recorded before the samples were autoclaved.

#### In vitro release studies

The in vitro release rates of the gels were measured using a test tube method described in previous studies [15]. 2 ml of prepared APS gel was transferred into tubes pre-weighted and placed in a 37°C water bath until a semi-solid gel was formed. After gelation, 4 ml of release medium (PBS, pH 7.4), pre-equilibrated to 37°C, was layered over the surface of the gel. The experiment was carried out at a constant temperature in a shaking water bath previously adjusted to 37 ± 0.2°C and 100 rev/min. At pre-determined time intervals, the entire release medium was removed and the weight of the remaining gel recorded. After weighing, 4 ml of the release medium was replaced by an equal volume of fresh medium. The percentage of gel weight loss was calculated by dividing the change in the gel weight by the initial gel weight. The gel dissolution test was conducted in triplicate.

#### Stability of APS in-situ gels

Three batches of APS in-situ gels were produced (01, 02 and 03). The physicochemical stability of APS in-situ gels was assessed at 4°C, 25°C and 40°C for 6 months. At various time points, the stability parameters, including appearance, *T* sol-gel, sol-gel transition time, pH, %LA labeled amount of APS, calculated based on glucose (C_6_H_12_O_4_)], and Viscosity of APS in-situ gels were determined as a function of storage time.

### In vivo test

#### Animals and drug administration

Male BALB/c mice (18–20 g) were purchased from Yangzhou University Laboratory Animal Center (Yangzhou, China). The mice were housed at 25 ± 1°C, with a 12 h light/12 h dark cycle and 50–60% relative humidity, with free access to food and water, and mice were monitored twice a day during the experiments. At experimental endpoint mice were anaesthetised by isoflurane inhalation, euthanized by cervical dislocation and tissues rapidly harvested for further analysis. All animals were sacrificed concomitantly at same time and no animals became severely ill or died at any time prior to the experimental endpoint.All procedures involving animal care were approved by the ethics committee of the Chinese Academy of Agricultural Sciences.

After adapting to their new environment for 1 week, these mice were randomly divided into 8 groups of 15 animals per group. Healthy mice in group I were used as a control and injected with saline solution for 10 days. Healthy mice of group II, III and IV were given saline solution from days 1 to 3. From days 4 to 10, group II (APS_-C_ group) injected with a commercial formulation of APS injection (250 mg/kg body weight). Group III (APS_-G1_ group) were given APS gel (1500 mg/kg) once, on the 4^th^ day. Group IV (APS_-G2_ group) were given APS gel (1000 mg/kg) twice, on the 4^th^ and 7^th^ days. From days 1 to 3, Groups V, VI, VII, and VIII were given Cytoxan (CTX, 80 mg/kg/d). From days 4 to10, Group V (CTX group) was treated with saline solution, and Group VI (CTX+APS_-C_ group) was injected with a commercial formulation of APS (250 mg/kg). Group VII (CTX+APS_-G1_ group) mice were given APS gel (1500 mg/kg) once, on the 4th day. Group VIII (CTX+APS_-G2_ group) animals were given APS gel (1000 mg/kg) twice, on the 4th and 7th days. The mice were given CTX via intraperitoneal injection, and the regimens of other drugs were administered via subcutaneous injection. Twenty-four hours after the last dose, the animals were weighed and sacrificed by decapitation. The spleen and thymus were excised and immediately weighed. The thymus and spleen indices were calculated according to the following formula: index (mg/g) = (weight of thymus or spleen) /body weight.

#### Lymphocytes proliferation assay

The spleens were aseptically removed from the mice using scissors and forceps in 0.1 M cold PBS, gently homogenized, and passed through a 40 μm nylon cell strainer to produce single-cell suspensions in accordance with a previously describe method [16, 17]. The erythrocytes in the cell mixture were washed by hypo-osmotic hemolysis, and the cells were resuspended to a final density of 5 × 10^6^ cells/ml in RPMI 1640 medium, supplemented with 10% newborn bovine serum (Invitrogen Corp., Carlsbad, CA,USA), 100 U/ml streptomycin, and 100 U/ml penicillin. Splenocytes (100 μl/well) were seeded into a 96-well plate containing ConA (1 μg/well) or LPS (2 μg/well). The spleen cells were then cultured for 3 days in a 5% CO_2_ atmosphere at 37°C, and then further incubated for 4.5 h with 10 μl MTT (5 mg/ml) per well. The plate was centrifuged at 200 × g for 15 min, and the supernatant was discarded. DMSO (100 μl) was added to each well, which was then shaken until all crystals dissolved. The absorbance at 570 nm was measured on a Multiskan MK3 microplate reader (ThermoFisher Scientific, Waltham, MA, USA).

#### Serum IL-2, IL-6, IgG and IgM assay

Serum was collected by eyeball enucleation 24 h after the last administration of CMP. The concentrations of IL-2, IL-6, IgG and IgM in the sera were determined using ELISA kits according to the manufacturer’s instructions.

### Statistical analysis

The data were analyzed using GraphPad Prism 5 and are presented as mean ± SD. The differences were considered significant if *P* < 0.05.

## Results

### Preparation of APS and APS in situ gels

In this study, APS was successfully purified from the roots of *radix astragali*. The yield of polysaccharide extracted from *radix astragali* was 10.17% of the raw material, and the polysaccharide concentration was 400 mg/ml in the APS extracting solution.

APS in-situ gels, prepared by the cold method, were transparent at all test temperatures (4 ± 1, 25 ± 1 and 35 ± 1°C) and formed stable and homogeneous gels at a suitable temperature.

This suggested that APS has good compatibility with poloxamer. The results of the content analysis, presented as %LA labeled amount of APS, calculated based on glucose (C_6_H_12_O_4_)], and shown in Table 2, suggest that all products could be accepted for further experiments for the APS content within the range of 100 ± 10% of labeled amount.

**Table 2:** Results of evaluation of thermoresponsive APS in-situ gels.

Formulation	*T* sol-gel(°C)	sol-gel transition time(s)		pH	% LA	Viscosity(mpa∙s,25°C)	Flow ability at temperatures (°C)		
		35(°C)	37(°C)				4 ± 1 (°C)	25 ± 1(°C)	35 ± 1(°C)
P407-16	38.2 ± 0.2	n.d.	n.d.	6.5	100.5 ± 0.8	n.d.	+++	+++	+++
P407-18	27.6 ± 0.3	n.d.	n.d.	6.4	101.6 ± 0.6	n.d.	+++	+	-
P407-20	17.1 ± 0.1	n.d.	n.d.	6.5	100.7 ± 0.5	n.d.	+++	-	-
P407-18+P188-1	29.7 ± 0.2	n.d.	n.d.	6.3	99.8 ± 0.4	n.d.	+++	+	-
P407-18+P188-1.5	32.5 ± 0.3	n.d.	n.d.	6.4	99.4 ± 0.3	n.d.	+++	++	-
P407-18+P188-2	34.1 ± 0.4	123.4 ± 1.7	98.6 ± 1.3	6.3	102.1 ± 0.9	36.8 ± 1.3	+++	+++	-
P407-18+P188-2.5	38.6 ± 0.2	n.d.	n.d.	6.3	101.3 ± 0.7	n.d.	+++	+++	+++
P407-18+P188-2+C-0.1	33.5 ± 0.5	37.9 ± 0.5	25.3 ± 0.2	6.5	100.2 ± 0.5	47.5 ± 1.7	+++	++	-
P407-18+P188-2+C-0.15	33.8 ± 0.3	20.2 ± 0.3	10.5 ± 0.2	6.5	101.4 ± 0.6	56.2 ± 1.6	+++	++	-
P407-18+P188-2+C-0.2	34.0 ± 0.3	13.6 ± 0.1	5.6 ± 0.1	6.5	101.4 ± 0.6	68.2 ± 2.1	+++	+	-

(n = 3,mean ± SD),% LA = labeled amount of APS, calculated based on glucose (C6H12O4), *T* sol-gel = sol-gel transition temperature, n.d. = not determined.

Flow ability: +++ = very good; ++ = good; + = average;− = no flow.

### In vitro characterization

#### The optimal dosage of ingredients in formulation

Gelation temperatures are considered suitable if they are in the range 33°C to 35°C, as the normal body temperature of mice is 37°C. As presented in Table 2, *T* sol-gel of P407-16 was 38.2 ± 0.2°C, which is higher than the body temperature of mice. *T* sol-gel of P407-18 and P407-20 were 27.6 ± 0.3 and 17.1 ± 0.1°C respectively, and thus P407-18 was selected as a prototype because it had a *T* sol-gel much closer to 34.0°C. The addition of P188 to P407-18 could raise *T* sol-gel of APS in-situ gels. P407-18 + P188-2 was selected as the best combination because it had a preferable *T* sol-gel(34.1 ± 0.4°C), as well as good flow ability. Table 2 points out that the addition of CMC-Na to P407-18 + P188-2 at concentrations of 0.1%, 0.15% and 0.2% does not alter *T* sol-gel of the products significantly, but increases the viscosity, shortening the sol-gel transition time.

#### In vitro release studies

**Fig 1:**
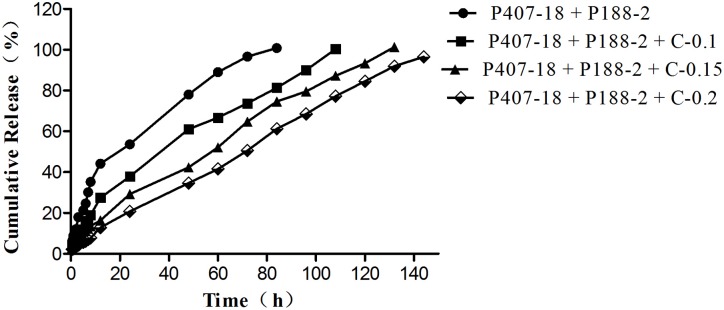
Release profiles of APS from in-situ forming gel formulations containing CMC-Na with different concentrations in PBS at 37°C (n = 3; error bars were omitted for clarity).

**Fig 2:**
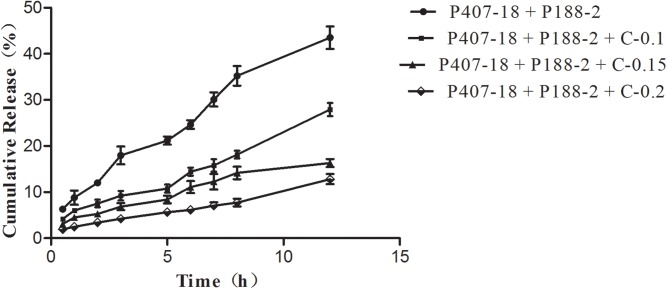
Release profiles of APS for the first 12 h from in-situ forming gel formulations containing CMC-Na with different concentrations in PBS at 37°C (n = 3; error bars were omitted for clarity).

**Fig 3:**
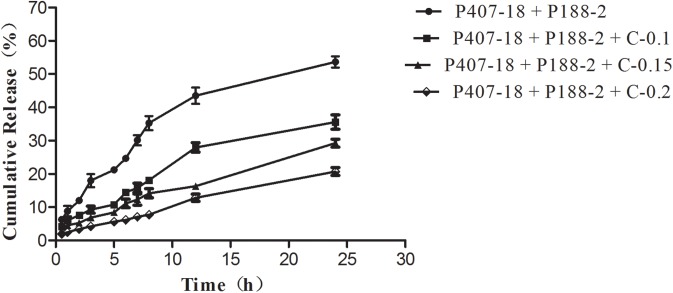
Release profiles of APS for the first 24h from in situ forming gel formulations containing CMC-Na with different concentrations in PBS at 37°C (n = 3; error bars were omitted for clarity).

The drug release from poloxamer-based formulations containing CMC-Na (0.1%, 0.15%, 0.2%) were analyzed relative to the control formulation containing certain amounts of P407 and P188 without CMC-Na (Fig 1). A decrease of initial burst release in the first 12 h (Fig 2) and 24 h (Fig 3) was observed in the release profiles of all formulations with CMC-Na compared with the control formulation, with a noticeable tendency for the initial burst release to decrease as CMC-Na increased. As high viscosity and poor fluidity are not suitable for injection, in-situ gels with 0.2% CMC-Na were excluded. Of the others, in-situ gels with 0.15% CMC-Na behaved best, having an initial release of about 16.30% in 12 h and 29.23% in 24 h, followed by sustained release of up to 100% within 132 h, while the control formulation had an initial release of about 44.16% in 12 h and 53.63% in 24 h, followed by release of up to 100% within 84 h.

The in vitro release study results indicate that the Ritger-Peppas models fitted well (y = 0.663x+1.281,R^2^ = 0.9930).

#### Autoclaving sterilization

The effect of autoclaving sterilization on the physicochemical properties of P407-18 + P188-2 + C-0.15 was determined. It was found that autoclaving sterilization did not significantly alter the viscosity, % LA, pH, or *T* sol-gelor time in any of the test samples, as seen in Table 3.

**Table 3:** Effect of autoclaving sterilization on physicochemical properties of APS in-situ gels.

	Lot number	T sol-gel (°C)	sol-geltransition time(s,37°C)	pH	%LA	Viscosity (mpa∙s, 25°C)
Before autoclaving	01	34.1 ± 0.2	10.3 ± 0.1	6.5	101.1 ± 0.9	56.8 ± 1.2
	02	33.6 ± 0.4	9.7 ± 0.1	6.4	100.5 ± 0.7	57.2 ± 1.3
	03	34.3 ± 0.3	10.6 ± 0.2	6.5	101.6 ± 1.1	55.9 ± 0.7
After autoclaving	01	33.9 ± 0.3	9.9 ± 0.1	6.4	100.8 ± 1.2	57.2 ± 1.0
	02	33.7 ± 0.5	9.6 ± 0.1	6.3	101.1 ± 0.9	56.6 ± 0.8
	03	34.1 ± 0.2	10.4 ± 0.1	6.4	102.0 ± 0.7	56.1 ± 0.9

(n = 3, mean ± SD), % LA = labeled amount of APS, calculated based on glucose (C6H12O4), T sol-gel = sol-gel transition temperature.

#### Storage stability

The stability of the APS in-situ gels was evaluated by monitoring the gels at 4°C, 25°C and 40°C for 6 months. Table 4 showed that APS in-situ gels had no precipitation and crystallization after the operation above. No remarkable variation for any parameter, including *T* sol-gel, sol-gel transition time, pH, %LA and Viscosity.

**Table 4:** Results of the stability test for APS in-situ gels.

Test item	4°C			25°C			40°C		
	01	02	03	01	02	03	01	02	03
Appearance	brown clear liquid	brown clear liquid	brown clear liquid	brown clear liquid	brown clear liquid	brown clear liquid	brown clear liquid	brown clear liquid	brown clear liquid
T sol-gel (°C)	34.0 ± 0.1	33.8 ± 02	34.1 ± 0.1	34.0 ± 0.2	33.9 ± 0.3	33.7 ± 0.2	34.2 ± 0.5	33.8 ± 0.1	33.7 ± 0.2
sol-gel transition time(s)	9.5± 0.1	10.3 ± 0.2	9.9 ± 0.1	9.6 ± 0.1	10.2 ± 0.1	9.8 ± 0.1	10.0 ± 0.1	9.9 ± 0.1	9.7 ± 0.1
pH	6.5	6.4	6.4	6.4	6.5	6.4	6.5	6.4	6.3
%LA	100.8 ± 1.2	100.2 ± 0.9	102.2 ± 0.7	101.0 ± 1.5	101.0 ± 1.3	101.3 ± 0.8	100.5 ± 1.1	100.4 ± 0.7	101.7 ± 0.6
Viscosity(mpa∙s, 25°C)	56.7 ±0.6	56.9 ± 0.5	57.0 ± 1.0	56.5 ±0.8	56.4 ± 0.8	57.5 ± 0.9	58.0 ±0.7	57.2 ± 0.7	57.8 ± 0.9

(n = 3, mean ± SD), % LA = labeled amount of APS, calculated based on glucose (C6H12O4), T sol-gel = sol-gel transition temperature.

### In vivo characterization

#### The dynamic changes of organ indices and lymphocytes proliferation

The changes in immune organ indices are listed in Table 5, and the A_570_ values in each group are shown in Table 6 The spleen and thymus indices and the spleen lymphocyte proliferation of the APS_-C_, APS_-G1_, and APS_-G2_ groups showed significantly higher values than those in the control group (*P* < 0.05), while those of the APS_-G1_ groups were consistent with those for the APS_-C_ group (*P* > 0.05). The indices and the spleen lymphocyte proliferation of the APS_-G2_ group had significantly higher values than those of the APS_-C_ group (*P* < 0.05). Those of CTX group showed significantly lower values than the control group (*P* < 0.05), while the values for the CTX+APS_-C_, CTX+APS_-G1_, and CTX+APS_-G2_ groups were significantly higher than the CTX group (*P* < 0.05). There were no significant differences between the CTX+APS_-C_, CTX+APS_-G1_, and CTX+APS_-G2_ groups.

**Table 5:** Changes of immune organ indices in each group.

Groups	Spleen index	Thymus index
Normal control	4.08 ± 0.18 ^ae^	1.84 ± 0.15 ^a^
APS_-C_	5.01 ± 0.31 ^b^	2.32 ± 0.09 ^bd^
APS_-G1_	5.02 ± 0.41 ^b^	2.22 ± 0.15 ^d^
APS_-G2_	5.93 ± 0.60 ^c^	2.63 ± 0.28 ^c^
CTX	2.80 ± 0.21 ^d^	1.03 ± 0.14 ^e^
CTX+APS_-C_	3.86 ± 0.43 ^ae^	1.70 ± 0.14 ^a^
CTX+APS_-G1_	3.80 ± 0.45 ^a^	1.64 ± 0.22 ^a^
CTX+APS_-G2_	4.85 ± 0.44 ^eb^	1.86 ± 0.20 ^a^

Column data without the same superscripts (a–e) differ significantly (*P* ˂ 0.05)($\bar{\text{x}}$ ± SD, n = 15/group).

**Table 6:** The cellular A570 values of every group in vivo test.

Groups	Con-A	LPS
Normal control	0.37 ± 0.02 ^a^	0.44 ± 0.03 ^af^
APS-C	0.45 ± 0.03 ^b^	0.52 ± 0.02 ^b^
APS-G1	0.42 ± 0.02 ^b^	0.49 ± 0.05 ^bf^
APS-G2	0.50 ± 0.04 ^c^	0.59 ± 0.04 ^c^
CTX	0.21 ± 0.02 ^d^	0.24 ± 0.02 ^d^
CTX+APS-C	0.32 ± 0.02 ^aef^	0.35 ± 0.03 ^eg^
CTX+APS-G1	0.28 ± 0.02 ^e^	0.32 ± 0.03 ^e^
CTX+APS-G2	0.34 ± 0.03 ^af^	0.40 ± 0.02 ^ag^

Column data without the same superscripts (a–g) differ significantly (*P* ˂ 0.05)($\bar{\text{x}}$ ± SD, n = 15/group).

#### Effects of APS in situ gel on serum IgM, IgG, IL-2, and IL-6 concentration

**Fig 4:**
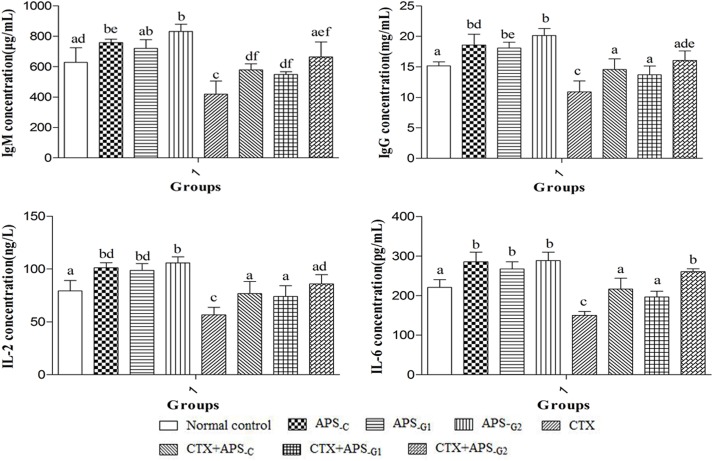
Changes in IgM, IgG, IL-2, and IL-6 concentrations in each group. a-c Data within a column without the same superscripts differ significantly (P < 0.05).

As shown in Fig 4, the serum IgM, IgG, IL-2, and IL-6 levels were significantly higher in the APS_-C_, APS_-G1_, and APS_-G2_ groups (*P* < 0.05), compared with those in the control group. However, there were no significant differences between the APS_-C_, APS_-G1_, and APS_-G2_ groups (*P* > 0.05). The serum IgM, IgG, IL-2, and IL-6 levels were significantly lower in the CTX group (*P* < 0.05), compared with those in the control group. However, notable enhancements in serum IgM, IgG, IL-2, and IL-6 levels were observed in the CTX+APS_-C_, CTX+APS_-G1_, and CTX+APS_-G2_ groups, compared to the CTX group (*P* < 0.05). In CTX+APS_-G1_ and CTX+APS_-G2_ group, the serum IgM, IgG, IL-2, and IL-6 concentration showed no significant changes, compared with those in the CTX+APS_-C_ group (*P* > 0.05).

## Discussion

The polysaccharide in the oral doses can be destroyed by enzymes, resulting in low bioavailability, so APS injection is widely used in veterinary clinics. For maximum immune-enhancing efficacy, the recommended use of APS injection was once a day for 4 to 7 consecutive days. In this study, we aimed to develop an effective and temperature-sensitive injectable in-situ forming APS gel, able to effect treatment with just 1 or 2 administrations, with an appropriate transition temperature (33~35°C), short gelation time, long delivery time, and relative ease of use. Therefore, we first screened and optimized the formulation and production of the gel. In this study, poloxamer 407 (P407) was used for the gel matrix. At low temperatures and concentrations, P407 exists in a single form in aqueous solution. However, when the temperature is higher than the critical micelle temperature (CMT), or the concentration of P407 reaches the critical micelle concentration (CMC) in aqueous solution, P407 exists as spherical, rod-like or layered micelles with the hydrophobic PPO as the core and the hydrophilic PEO as the shell. Furthermore, when the temperature increases to the phase-transition temperature, the micelles are closely packed and form a semi-solid gel. Therefore, the higher the concentration of P407, the more prone the solution is to gel-forming phase transition [8, 9]. Similarly, the gel-forming temperature of the APS gel decreased as its P407 concentration increased. The 18% P407 system was preferred, because its gel temperature (27.6 ± 0.3°C) was closer to the ideal temperature than that of the 16% P407 mix (38.2 ± 0.2°C) or 20% P407 mix (17.1 ± 0.1°C). In this study, Poloxamer 188 (P188) is used as a temperature regulator, and the gelling temperature increases with the P188 concentration in the solution [8]. However, it is reported that addition of too high P188 concentration into P407 solution might decrease sol—gel transition temperature of the products due to increase in effective concentration of F68 [13]. Therefore, the optimum of P188 concentration was considered for optimization in this study. And the P407-18+P188-2 was found to be optimal, being less viscous at room temperature and easy to inject.

In APS gels, CMC-Na is added to increase the gel’s strength, reduce the incidence of drug burst release, and prolong the time of release [18]. In view of the requirement for ease of administration, 0.15% CMC-Na was selected as the best concentration. The addition of 0.15% CMC-Na resulted in extensions of release times to 132 h. The release study indicate that the Ritger-Peppas models fitted well (y = 0.663x+1.281,R^2^ = 0.9930). In general, Two main factors contributed to drug release from P407 based formulation. The first is the diffusion of drug molecules through the water channels present in gel structure, and the second is the drug release concurrent with the dissolution of the P407 gel matrix. APS in-situ gels was a solution preparation, and APS dissolve easily in water. APS could dissolve in release medium (PBS, pH 7.4) with the dissolution of the P407 gel matrix.

Three batches of samples were prepared according to the optimal formulation. After high pressure steam sterilization at 121°C for 20 minutes, the characteristics of the three batches were no different to before sterilization. Therefore, they all qualified as having good repeatability.

In order further to confirm the effect of its slow release, the normal mice and the CTX-induced immune suppressed mice were given APS in-situ gel (on 1 or 2 occasions each) in this study. The representative indices of immune function, such as immune organ index, spleen lymphocyte proliferation, immune globulin, and levels of immune-related cytokines were used to evaluate immune system enhancement of the in-situ forming gel. The spleen and thymus are the main sites of immune cell growth and proliferation, which directly affect the immune function of the body [19]. In this study, the spleen and thymus indices of mice increased significantly after they were given APS in-situ gel 1 or 2 times (Table 5), which was consistent with that of mice given daily APS injections (7 times). T lymphocytes are the key protagonists in cellular immune function, and B cells act in humoral immunity. LPS and ConA can both stimulate the proliferation of lymphocytes, with ConA acting on T cells and LPS on B cells. An assay of ConA and LPS induction a proliferation of spleen lymphocyte levels is often used to investigate the effect of drugs on B and T lymphocytes [20]. The results (Table 6) show that the production of T cells and B cells can be significantly accelerated using APS in-situ gel just once, which is the same as the effect induced by daily APS injections. In addition, the effect of two injections of APS in-situ gel was better that of daily APS injections. APS can promote the production of major immune globulins IgG and IgM, mediating complement activation, immune regulation, and other immune processes [21]. Additionally, APS promotes the release of cytokines such as IL-2 and IL-6, which can indirectly promote the proliferation of T cells and B cells and the production of immune globulin [22–24]. In this study, daily APS injections and in-situ gel injection on just 1 or 2 occasions can result in the same levels of IgM, IgG, IL-2 and IL-6. In the case of IL-6 in-situ gel injection had a better effect than the high-frequency injections. In vivo test results showed that the immuno-enhancement brought about by a single APS in-situ gel injection was consistent with that of APS injections administered daily for 7 days. Meanwhile, the positive effect on some indices of the in-situ gel was significantly greater than that induced by 7 daily injections. The in vivo release rate was consistent with that in vitro, with the in-situ gel releasing its payload slowly.

## Conclusion

In summary, a novel in-situ forming gel based on poloxamer composite matrix depot was developed as a sustained-elease depot for APS. The optimal formulation and process are as follows: 10 g Astragalus polysaccharide, 18 g P407, 2 g P188, 0.15 g CMC-Na, 0.85 g sodium chloride in 100 ml gel in situ. This gel system is prepared through the cold solution method and high pressure steam sterilization at 121°C for 20 minutes and is characterized by a suitable transition temperature, rapid phase change, ease of use, and an extended drug release in vitro up to 5.5 d. A single injection of the in-situ forming gel had an equivalent immune-boosting effect to a 7-day course of daily injections, while a single additional injection of the in-situ gel added yet further enhancements over high-frequency administration. Based on these results, it can be concluded that the poloxamer composite matrix depot is a promising carrier for the sustained release of APS with an ideal release behavior.

## Supporting information

S1 Table Formulation compositions in thermoresponsive APS in-situ gels. (DOCX)

S2 Table Results of evaluation of thermoresponsive APS in-situ gels. (DOCX)

S3 Table Effect of autoclaving sterilization on physicochemical properties of APS in-situ gels. (DOCX)

S4 Table Results of the stability test for APS in-situ gels. (DOCX)

S5 Table Changes of immune organ indices in each group. (DOCX)

S6 Table The cellular A570 values of every group in vivo test. (DOCX)
