# Supplementary material for: Optimization and evaluation of astragalus polysaccharide injectable thermoresponsive in-situ gels
Source: PLoS One. 2017 Mar 28;12(3):e0173949. doi: 10.1371/journal.pone.0173949 (PMC5369758; doi:10.1371/journal.pone.0173949)
Supplement: S1 Table — (DOCX) [file pone.0173949.s001.docx]

**Table1. Formulation compositions in thermoresponsive APS in-situ gels**

| **Content of ingredients in each formulation(%, w/v)** | **Ingredients** | | | | | |
| --- | --- | --- | --- | --- | --- | --- |
|  | **APS** | **P407** | **P188** | **CMC-Na** | **NaCl** | **Water for injection ad** |
| P407-16 | 10 | 16 | 0 | 0 | 0.85 | 100 |
| P407-18 | 10 | 18 | 0 | 0 | 0.85 | 100 |
| P407-20 | 10 | 20 | 0 | 0 | 0.85 | 100 |
| P407-18+P188-1 | 10 | 18 | 1 | 0 | 0.85 | 100 |
| P407-18+P188-1.5 | 10 | 18 | 1.5 | 0 | 0.85 | 100 |
| P407-18+P188-2 | 10 | 18 | 2 | 0 | 0.85 | 100 |
| P407-18+P188-2.5 | 10 | 18 | 2.5 | 0 | 0.85 | 100 |
| P407-18+P188-2+C-0.1 | 10 | 18 | 2 | 0.1 | 0.85 | 100 |
| P407-18+P188-2+C-0.15 | 10 | 18 | 2 | 0.15 | 0.85 | 100 |
| P407-18+P188-2+C-0.2 | 10 | 18 | 2 | 0.2 | 0.85 | 100 |
