# Supplementary material for: Optimization and evaluation of astragalus polysaccharide injectable thermoresponsive in-situ gels
Source: PLoS One. 2017 Mar 28;12(3):e0173949. doi: 10.1371/journal.pone.0173949 (PMC5369758; doi:10.1371/journal.pone.0173949)
Supplement: S2 Table — (DOCX) [file pone.0173949.s002.docx]

**Table2. Results of evaluation of thermoresponsive APS in-situ gels**

| **Formulation** | ***T* sol-gel(℃)** | **sol-gel transition time(s)** | | **pH** | **% LA** | **Viscosity (mpa∙s,25℃)** | **Flow ability at temperatures (℃)** | | |
| --- | --- | --- | --- | --- | --- | --- | --- | --- | --- |
|  |  | **35(℃)** | **37(℃)** |  |  |  | **4 ± 1 (℃)** | **25 ± 1(℃)** | **35 ± 1(℃)** |
| P407-16 | 38.2 ± 0.2 | n.d. | n.d. | 6.5 | 100.5 ± 0.8 | n.d. | +++ | +++ | +++ |
| P407-18 | 27.6 ± 0.3 | n.d. | n.d. | 6.4 | 101.6 ± 0.6 | n.d. | +++ | + | - |
| P407-20 | 17.1 ± 0.1 | n.d. | n.d. | 6.5 | 100.7 ± 0.5 | n.d. | +++ | - | - |
| P407-18+P188-1 | 29.7 ± 0.2 | n.d. | n.d. | 6.3 | 99.8 ± 0.4 | n.d. | +++ | + | - |
| P407-18+P188-1.5 | 32.5 ± 0.3 | n.d. | n.d. | 6.4 | 99.4 ± 0.3 | n.d. | +++ | ++ | - |
| P407-18+P188-2 | 34.1 ± 0.4 | 123.4 ± 1.7 | 98.6 ± 1.3 | 6.3 | 102.1 ± 0.9 | 36.8 ± 1.3 | +++ | +++ | - |
| P407-18+P188-2.5 | 38.6 ± 0.2 | n.d. | n.d. | 6.3 | 101.3 ± 0.7 | n.d. | +++ | +++ | +++ |
| P407-18+P188-2+C-0.1 | 33.5 ± 0.5 | 37.9 ± 0.5 | 25.3 ± 0.2 | 6.5 | 100.2 ± 0.5 | 47.5 ± 1.7 | +++ | ++ | - |
| P407-18+P188-2+C-0.15 | 33.8 ± 0.3 | 20.2 ± 0.3 | 10.5 ± 0.2 | 6.5 | 101.4 ± 0.6 | 56.2 ± 1.6 | +++ | ++ | - |
| P407-18+P188-2+C-0.2 | 34.0 ± 0.3 | 13.6 ± 0.1 | 5.6 ± 0.1 | 6.5 | 101.4 ± 0.6 | 68.2 ± 2.1 | +++ | + | - |

(n = 3,mean ± SD),% LA = labeled amount of APS, calculated based on glucose (C6H12O4), *T* sol-gel = sol-gel transition temperature, n.d. = not determined.

Flow ability: +++ = very good; ++ = good; + = average; - = no flow.
