# Supplementary material for: Optimization and evaluation of astragalus polysaccharide injectable thermoresponsive in-situ gels
Source: PLoS One. 2017 Mar 28;12(3):e0173949. doi: 10.1371/journal.pone.0173949 (PMC5369758; doi:10.1371/journal.pone.0173949)
Supplement: S3 Table — (DOCX) [file pone.0173949.s003.docx]

**Table3. Effect of autoclaving sterilization on physicochemical properties of APS in-situ gels.**

|  | **Lot number** | **T sol-gel (℃)** | **sol-geltransition time(s,37℃)** | **pH** | **%LA** | **Viscosity (mpa∙s, 25℃)** |
| --- | --- | --- | --- | --- | --- | --- |
| Before autoclaving | 01 | 34.1 ± 0.2 | 10.3 ± 0.1 | 6.5 | 101.1 ± 0.9 | 56.8 ± 1.2 |
|  | 02 | 33.6 ± 0.4 | 9.7 ± 0.1 | 6.4 | 100.5 ± 0.7 | 57.2 ± 1.3 |
|  | 03 | 34.3 ± 0.3 | 10.6 ± 0.2 | 6.5 | 101.6 ± 1.1 | 55.9 ± 0.7 |
| After autoclaving | 01 | 33.9 ± 0.3 | 9.9 ± 0.1 | 6.4 | 100.8 ± 1.2 | 57.2 ± 1.0 |
|  | 02 | 33.7 ± 0.5 | 9.6 ± 0.1 | 6.3 | 101.1 ± 0.9 | 56.6 ± 0.8 |
|  | 03 | 34.1 ± 0.2 | 10.4 ± 0.1 | 6.4 | 102.0 ± 0.7 | 56.1 ± 0.9 |

(n = 3, mean ± SD), % LA = labeled amount of APS, calculated based on glucose (C_6_H_12_O_4_), T sol-gel = sol-gel transition temperature.
