# Supplementary material for: Optimization and evaluation of astragalus polysaccharide injectable thermoresponsive in-situ gels
Source: PLoS One. 2017 Mar 28;12(3):e0173949. doi: 10.1371/journal.pone.0173949 (PMC5369758; doi:10.1371/journal.pone.0173949)
Supplement: S4 Table — (DOCX) [file pone.0173949.s004.docx]

**Table4. Results of the stability test for APS in-situ gels.**

| **Test item** | **4℃** | | | **25℃** | | | **40℃** | | |
| --- | --- | --- | --- | --- | --- | --- | --- | --- | --- |
|  | **01** | **02** | **03** | **01** | **02** | **03** | **01** | **02** | **03** |
| Appearance | brown clear liquid | brown clear liquid | brown clear liquid | brown clear liquid | brown clear liquid | brown clear liquid | brown clear liquid | brown clear liquid | brown clear liquid |
| T sol-gel (℃) | 34.0 ± 0.1 | 33.8 ± 02 | 34.1 ± 0.1 | 34.0 ± 0.2 | 33.9 ± 0.3 | 33.7 ± 0.2 | 34.2 ± 0.5 | 33.8 ± 0.1 | 33.7 ± 0.2 |
| sol-gel transition time(s) | 9.5± 0.1 | 10.3 ± 0.2 | 9.9 ± 0.1 | 9.6 ± 0.1 | 10.2 ± 0.1 | 9.8 ± 0.1 | 10.0 ± 0.1 | 9.9 ± 0.1 | 9.7 ± 0.1 |
| pH | 6.5 | 6.4 | 6.4 | 6.4 | 6.5 | 6.4 | 6.5 | 6.4 | 6.3 |
| %LA | 100.8 ± 1.2 | 100.2 ± 0.9 | 102.2 ± 0.7 | 101.0 ± 1.5 | 101.0 ± 1.3 | 101.3 ± 0.8 | 100.5 ± 1.1 | 100.4 ± 0.7 | 101.7 ± 0.6 |
| Viscosity(mpa∙s, 25℃) | 56.7 ±0.6 | 56.9 ± 0.5 | 57.0 ± 1.0 | 56.5 ±0.8 | 56.4 ± 0.8 | 57.5 ± 0.9 | 58.0 ±0.7 | 57.2 ± 0.7 | 57.8 ± 0.9 |

(n = 3, mean ± SD), % LA = labeled amount of APS, calculated based on glucose (C_6_H_12_O_4_), T sol-gel = sol-gel transition temperature
