# Supplementary material for: Optimization and evaluation of astragalus polysaccharide injectable thermoresponsive in-situ gels
Source: PLoS One. 2017 Mar 28;12(3):e0173949. doi: 10.1371/journal.pone.0173949 (PMC5369758; doi:10.1371/journal.pone.0173949)
Supplement: S5 Table — (DOCX) [file pone.0173949.s005.docx]

**Table5. Changes of immune organ indices in each group.**

| **Groups** | **Spleen index** | **Thymus index** |
| --- | --- | --- |
| Normal control | 4.08 ± 0.18 ^ae^ | 1.84 ± 0.15 ^a^ |
| APS_-C_ | 5.01 ± 0.31 ^b^ | 2.32 ± 0.09 ^bd^ |
| APS_-G1_ | 5.02 ± 0.41 ^b^ | 2.22 ± 0.15 ^d^ |
| APS_-G2_ | 5.93 ± 0.60 ^c^ | 2.63 ± 0.28 ^c^ |
| CTX | 2.80 ± 0.21 ^d^ | 1.03 ± 0.14 ^e^ |
| CTX+APS_-C_ | 3.86 ± 0.43 ^ae^ | 1.70 ± 0.14 ^a^ |
| CTX+APS_-G1_ | 3.80 ± 0.45 ^a^ | 1.64 ± 0.22 ^a^ |
| CTX+APS_-G2_ | 4.85 ± 0.44 ^eb^ | 1.86 ± 0.20 ^a^ |

Column data without the same superscripts (a–e) differ significantly (*P* ˂ 0.05)($\overline{\text{x}}$± SD, n = 15/group).
