# Supplementary material for: Optimization and evaluation of astragalus polysaccharide injectable thermoresponsive in-situ gels
Source: PLoS One. 2017 Mar 28;12(3):e0173949. doi: 10.1371/journal.pone.0173949 (PMC5369758; doi:10.1371/journal.pone.0173949)
Supplement: S6 Table — (DOCX) [file pone.0173949.s006.docx]

**Table6. The cellular A570 values of every group in vivo test.**

| **Groups** | **Con-A** | **LPS** |
| --- | --- | --- |
| Normal control | 0.37 ± 0.02 ^a^ | 0.44 ± 0.03 ^af^ |
| APS-C | 0.45 ± 0.03 ^b^ | 0.52 ± 0.02 ^b^ |
| APS-G1 | 0.42 ± 0.02 ^b^ | 0.49 ± 0.05 ^bf^ |
| APS-G2 | 0.50 ± 0.04 ^c^ | 0.59 ± 0.04 ^c^ |
| CTX | 0.21 ± 0.02 ^d^ | 0.24 ± 0.02 ^d^ |
| CTX+APS-C | 0.32 ± 0.02 ^aef^ | 0.35 ± 0.03 ^eg^ |
| CTX+APS-G1 | 0.28 ± 0.02 ^e^ | 0.32 ± 0.03 ^e^ |
| CTX+APS-G2 | 0.34 ± 0.03 ^af^ | 0.40 ± 0.02 ^ag^ |

Column data without the same superscripts (a–g) differ significantly (*P* ˂ 0.05)($\overline{\text{x}}$± SD, n = 15/group).
